# Method Development for the Prediction of Melt Quality in the Extrusion Process

**DOI:** 10.3390/polym16091197

**Published:** 2024-04-25

**Authors:** Dorte Trienens, Volker Schöppner, Peter Krause, Thomas Bäck, Seraphin Tsi-Nda Lontsi, Finn Budde

**Affiliations:** 1 Kunststofftechnik Paderborn, Paderborn University, 33098 Paderborn, Germany; volker.schoeppner@ktp.upb.de (V.S.); finnluk@web.de (F.B.); 2 Divis Intelligent Solutions GmbH, 44227 Dortmund, Germany; krause@divis-gmbh.de (P.K.); lontsi@divis-gmbh.de (S.T.-N.L.); 3 Leiden Institute of Advanced Computer Science (LIACS), Leiden University, Einsteinweg 55, 2333 Leiden, The Netherlands

**Keywords:** polymer extrusion process, screw performance index, melt quality, simulation, machine learning

## Abstract

Simulation models are used to design extruders in the polymer processing industry. This eliminates the need for prototypes and reduces development time for extruders and, in particular, extrusion screws. These programs simulate, among other process parameters, the temperature and pressure curves in the extruder. At present, it is not possible to predict the resulting melt quality from these results. This paper presents a simulation model for predicting the melt quality in the extrusion process. Previous work has shown correlations between material and thermal homogeneity and the screw performance index. As a result, the screw performance index can be used as a target value for the model to be developed. The results of the simulations were used as input variables, and with the help of artificial intelligence—more precisely, machine learning—a linear regression model was built. Finally, the correlation between the process parameters and the melt quality was determined, and the quality of the model was evaluated.

## 1. Introduction

In the polymer industry, polymers are mainly processed on injection molding machines and extruders [1]. Cost efficiency plays an important role in these manufacturing processes [2]. Especially in the field of extrusion, high throughput rates must be achieved in order to maximize profits while maintaining the specified quality requirements of the products [3]. The cost efficiency of the extrusion process depends on many factors. In addition to the process parameters (screw speed, barrel temperatures), the plasticizing and homogenizing performance of the extruder plays a central role [4]. For these reasons, the optimum extruder design, and in particular the design of the extruder screw, is the goal of every extruder engineer [5]. The manufacture of extruder screws is very time-consuming and costly, which is why the use of prototypes is avoided. Therefore the design of the screw is based on experience and simulations [6]. One example is the simulation software developed at Kunststofftechnik Paderborn (KTP), which uses an iterative approach to simulate the entire extrusion process inside the extruder and, for example, outputs pressure and temperature curves over the entire screw length [7]. This simulation software does not currently provide a qualitative evaluation of the simulation results regarding the resulting melt quality. Up to now, product quality can only be determined in complex experimental tests after the production process.

There are numerous definitions of homogeneous melt quality in the literature because there is no established test for determining melt quality. According to DIN EN ISO 9000:2015-11, quality is defined as “the degree to which a set of inherent characteristics of an object satisfies requirements” [8]. In this case, the requirements for a plastic melt describe the ability to extrude a product that meets the specified requirements. Therefore, there is a direct correlation between product quality and melt quality. Gorczyca described a consistent material quality due to a pulseless throughput, a materially and thermally homogeneous melt with simultaneously low melt temperature [9]. Choosing the right process settings, such as screw speed and barrel temperatures, is also critical to melt quality [10]. When the temperature is increased at constant pressure, the viscosity of the melt decreases. If the material is overheated, the material properties change or the melt decomposes, reducing product quality [11]. Therefore, the temperature and its distribution across the screw channel have a direct effect on the quality and properties of the product [12]. There are several approaches to evaluating thermal and material homogeneity. For the investigations in this research work, the thermal homogeneity of the melt was assessed using the standard deviation of the weighted average melt temperature at the screw tip. A high standard deviation indicates low thermal homogeneity [13]. Dead stop tests were performed to determine material homogeneity. In dead-stop tests, the stationary extrusion process is abruptly stopped, and the melt is cooled. The screw is then retracted, and samples can be taken. Black masterbatch was added for this purpose. The black material is mixed into the natural-colored base material at a certain percentage rate. Thin sections were taken at the screw tip and evaluated by gray scale analysis. Again, the standard deviation of the gray value was used to evaluate material homogeneity, as described in [14].

In the previous research work, melt quality and possible correlations between process parameters were investigated. The investigations showed that minimal geometric differences in the screw concepts had a negligible influence on the melt quality, so that an extensive investigation plan can be dispensed with. This means that a model for melt quality can be established independently of screw geometry [15]. Correlations between thermal and material homogeneity and the Screw Performance Index (SPI) developed by Dörner were also investigated. The SPI has been found to correlate with both thermal and material homogeneity. If the melt is inhomogeneous, the standard deviation of the weighted average melt temperature and the standard deviation of the gray value increase. At the same time, an inhomogeneous melt results in a low SPI [16].

### 1.1. Screw Performance Index (SPI)

The SPI can be used to make a qualified statement about the melt quality in the experiment. Several process characteristics were recorded and evaluated. These include throughput, degree of melting, melt temperature, thermal homogeneity, pressure above the screw, pressure and temperature fluctuations at the screw tip, material damage, and energy efficiency. Equation (1) shows the calculation of the SPI. The SPI evaluates the entire extrusion process using a number between zero (bad process) and one (very good process). In addition, two necessary conditions are defined that, if not met, directly define a SPI of “0” for that process point. One of them is the degree of melting. For this purpose, a transparent film is extruded and evaluated by at least two different persons for visible defects, such as unmelted particles or air inclusions, and documented with a photo for later comparison of the operating points. The SPI is only rated if the film is completely melted (category rating “0”) or if a single sporadic defect occurs within a maximum of one minute (category rating “1”); otherwise, the operating point is rated with an SPI of “0”. Second, the weighted average melt temperature must be within the processing temperatures specified by the manufacturer. Using this index, it is possible to make a statement about the melt quality during or shortly after the extrusion process without having to use complex evaluation methods [4].

(1) $$SPI=\left(-\frac{C_{k_{melt}}+C_{k_{{∆p}_{stern}}}+0.5\times C_{k_{Druck}}+C_{T_{GM}}+C_{k_{T_{GM}}}+C_{k_{tH}}+C_{k_{Eff}}+C_{k_{MFR}}}{7.5\times 4}+1\right),$$

$$C_{k_{melt}}>2\Rightarrow SPI=0$$

$$T_{GM}<T_{{Grenz}_{min}}\vee T_{GM}>T_{{Grenz}_{max}}\Rightarrow SPI=0$$

*SPI*: Screw performance index;
$T_{Grenz}$: Minimum and maximum processing temperatures of the polymer;
$C_{k_{melt}}$: Degree of melting;
$C_{k_{{∆p}_{stern}}}$: Back pressure fluctuation;
$C_{k_{Druck}}$: Difference between the max. pressure and the back pressure;
$C_{T_{GM}}$: Melt temperature;
$C_{k_{T_{GM}}}$: Temperature fluctuation;
$C_{k_{tH}}$: Thermal homogeneity;
$C_{k_{Eff}}$: Degree of efficiency;
$C_{k_{MFR}}$: Change in the melt flow rate.

Investigations of the correlations between thermal and material homogeneity and SPI confirm that there is a strong correlation between quality and SPI. The SPI is also very sensitive to variations in the standard deviation of weighted average melt temperature and standard deviation of the gray value. As the standard deviation increases, homogeneity deteriorates and the SPI decreases, and vice versa. The correlation between SPI and melt quality eliminates the need for time-consuming melt quality evaluations. Only random samples need to be taken to verify the results [16].

Since the SPI evaluates melt quality using a metric, it is an ideal target variable for the analytical model to simulate melt quality.

### 1.2. Simulation of the Extrusion Process

The goal of this research is the development of a model that will provide a qualitative indicator of the expected melt quality from the simulation results. Numerical simulations of plasticizing extruders are very limited and time consuming due to the presence of multiphase flow due to plastic melt (liquid) and pellets (solid) in the melt zone. For this reason, modeling and numerical simulations that describe the melt properties during processing are not state of the art.

An alternative to numerical simulation is the analytical calculation model. The REX program (computer-aided extruder design), which has been continuously developed at Kunststofftechnik Paderborn (KTP) since 1988, can be used for this purpose. The goal of REX is to simulate extrusion processes on workstations as realistically as possible, but also as quickly as possible, so that the calculation can be performed without a high-performance computer. For this reason, REX was and is developed on the basis of six principles [17]:

- The principle of the simple model.
- The principle of mathematical clarity of model solutions.
- The principle of mathematically closed solutions, even if rigorous simplifications are necessary.
- The principle of linearization of intermediate results in semi-logarithmic or double-logarithmic representation in order to obtain a closed solution for a sequence of differential equations to obtain a closed solution.
- The principle of approximation of numerical solutions when exact solutions are no longer possible.
- The principle of describing stochastic processes as well as possible by well-defined distribution functions.

**Figure 1:**
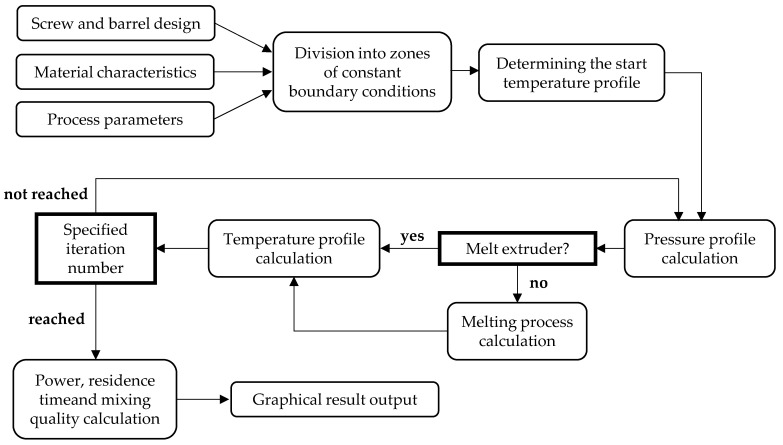
Internal calculation process of REX [7].

Figure 1 shows a schematic representation of the internal calculation process in REX. The operator only needs to enter the barrel and screw data, material properties, and process parameters for a complete calculation.

The screw is automatically divided into at least 150 intervals for calculation, and constant boundary conditions are set for each interval. By dividing the complex screw geometry into intervals of constant geometry, each interval can be calculated individually, and the results of all intervals can then be combined. In this way, the entire extrusion process in the extruder can be simulated using an iterative procedure [7].

Due to the comprehensive calculation in REX (e.g., pressure, temperature, melt and shear rate curves), the simulation results are useful for many applications in both industrial and scientific fields. The advantage is the short calculation time of a few seconds compared to the calculation times of numerical simulations, which can take up to several days. The melting calculation in the REX program is based on this modified Tadmor model, which takes into account the location-dependent melt film thickness on the cylinder wall. The leakage current across the bars is also included [17]. The simulation results considered in this work are maximum pressure, melt vortex formation, melt rate, melt temperature, drive power, minimum and average residence time, and back pressure.

### 1.3. Machine Learning (Comparison Classification and Regression)

Artificial intelligence (AI) is playing an increasingly important role and is being used in more and more areas, especially to increase efficiency. The term “machine learning” generally refers to a set of methods that use algorithmic learning processes to identify correlations in existing data sets in order to make predictions based on them [18]. The ability of a machine or software to learn certain tasks is based on the data on which it is trained [19]. There are three basic types of machine learning: supervised learning, unsupervised learning, and reinforcement learning [20]. The selection of the method to be used is influenced by the given problem and the available data sets. The data considered for this problem are the simulated process parameters from the simulation software and the experimentally determined SPI of the process parameters. Since these data have a known structure, there is no need for unsupervised learning, which uses unlabeled data with an unknown structure [18]. Reinforcement learning is a dynamic process in which the system continuously reacts to the current state of the environment and adapts accordingly [20]. However, the given data set on which the algorithm is to be trained is static and not time dependent. Therefore, this approach is not suitable either. The given data set contains the desired output values, the SPI, which were determined experimentally. This is called labeled data. The category of supervised learning includes algorithms that are trained with a large amount of labeled data in order to be able to make decisions independently. Since labeled data are given in the data set of the problem, supervised learning can be optimally applied. The goal of supervised learning is to learn a model using training data to make predictions about unknown or future data. In this case, this means being able to simulate the SPI for new process parameters. In this context, the term “supervised” means that the training data are already labeled with the desired output values [18,20]. Subcategories of supervised learning include regression and classification, which are discussed briefly below.

**Figure 2:**
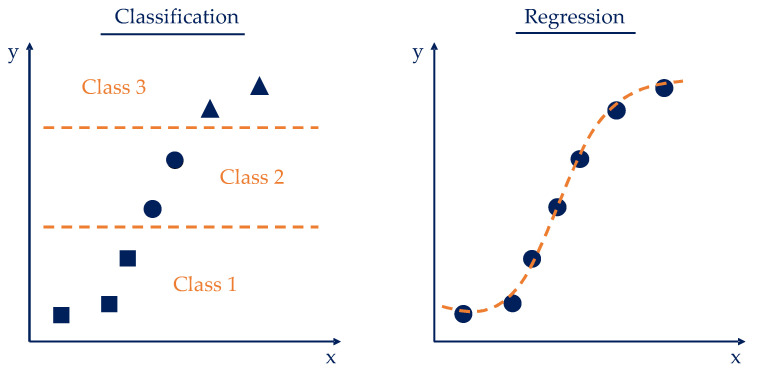
Comparison of classification (**left**) and regression (**right**) following [19].

The goal of classification is to predict the categorial classes of new instances based on previous observations. The names of these classes correspond to unique, unordered values that represent the group membership of the instances. Instances are then assigned categorical, unordered class labels. In regression analysis, various regressor variables (independent or explanatory variables) and a continuous target variable, the outcome, are specified. Based on this, a relationship is established between the variables in order to make predictions. The difference between classification and regression is that in regression, the output values are continuous and can therefore be functionally approximated [20]. Figure 2 shows a schematic visualization of how the classification and regression methods behave on an exemplary data set.

Due to the continuous output values in the given use case, there is a regression problem. Regression assumes that the data values *Y* come from different distributions, with a different distribution for each specific value *x* of the general variable *X*. This model is expressed symbolically in Equation (2):

(2) Y|X=x~p(y|x)

The expression *p*(*y*|*x*) represents the distribution of potentially observable values *Y* when *X* = *x*. It is referred to as the conditional distribution of *Y* under the condition that *X* = *x* [21]. In order to build a predictive model using machine learning, and supervised learning in particular, the types of data used must be taken into account, as it has a significant impact on the quality of the resulting predictive model [18].

## 2. Experimental Setup

**Figure 3:**
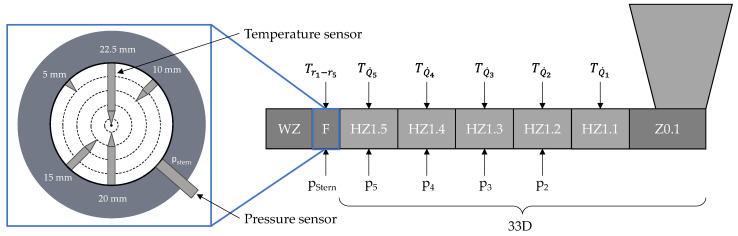
Schematic structure of the extruder and the measuring flange.

The data for the dataset used here were derived from the results of the experimental investigations and the corresponding simulations with REX. The experimental tests were carried out on a single screw extruder from Reifenhäuser Extrusion Systems GmbH (Troisdorf, Germany), model RH034-45-28D/HS, with a diameter of 45 mm and a barrel length of 33 D. The extruder has a cooled feed zone (Z0.1) and five heating zones (HZ1.1–HZ1.5). The barrel is equipped with temperature and pressure sensors along its entire length (T_Q1_–T_Q5_, p_2_–p_5_). A measuring flange (F) is mounted at the end of the extruder and contains five temperature sensors (T_r1_–T_r5_) that measure the temperature at different depths in the channel. The melt pressure (p_stern_) is also measured. The die is a flat film die (WZ) from COLLIN Lab and Pilot Solutions GmbH, Maitenbeth, Germany. Figure 3 shows the schematic structure of the extruder and the measuring flange. The signal was recorded at a sampling frequency of 10 Hz as this allows the temperature and pressure fluctuations to be mapped with sufficient accuracy.

Two different polymers from the company LyondellBasell (Rotterdam, The Netherlands) were used for the experimental investigations. The low-density polyethylene (LDPE) is called Lupolen 2420D, and the name of the polypropylene is HP420M. For the investigations of material homogeneity, color master batches were added to the base material. The Black 335 PP pigment was compounded into Moplen 420M from Argus Additive Plastics GmbH (Büren, Germany) and the Hostalen CRP 100 Black was compounded into the Lupolen 2420D. In order to compare the results of the grey value analysis, 0.4% of the pigment by mass was added to the PP and 7.5% by mass was added to the PE.

Three different screw designs, each with two different geometries, were used for the investigations. Two three-section screws, two barrier screws, and a standard three-section screw with two different shear and mixing section combinations were studied. The experimental set-up and investigations are described in detail in [15,16,22].

## 3. Method

The following section explains the methods used to develop the melt quality prediction model known as the Simulated Screw Performance Index (SSPI).

### 3.1. PPDAC

**Figure 4:**
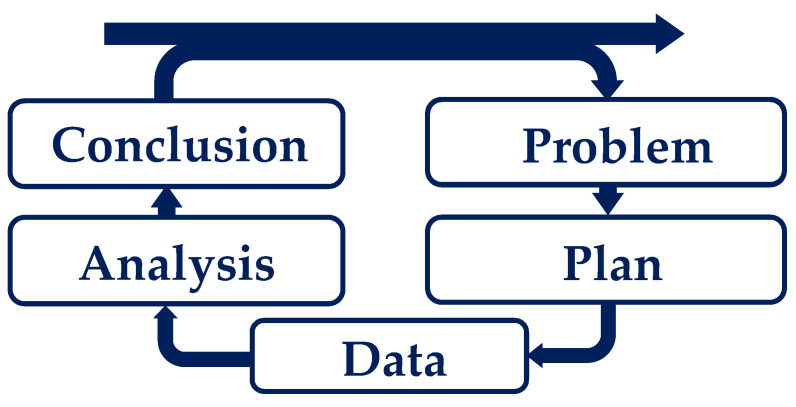
PPDAC model according to [24].

In order to simulate melt quality assessment in the extrusion process, a structured approach to develop a predictive model is required. For this reason, the PPDAC model (Problem, Plan, Data, Analysis, Conclusion) according to Wild and Pfannkuch [23] was used as a basis and combined with machine learning steps. The PPDAC model provides a proven framework for approaching the task systematically and performing the necessary steps in a logical sequence. The cycle shown in Figure 4 describes the approach to a data-driven problem. The individual phases of the process are specified to ensure a methodical approach and sound analysis to develop a simulative model on a given database, reflect on it, and make it available for further use cases through systematic documentation [24].

In the following, the different aspects of the PPDAC model are linked to the machine learning steps.

1. According to [23,25], the Problem analysis phase focuses on identifying the system dynamics and understanding and defining the problem. The following question can be used as a problem definition: How can melt quality be predicted by simulation? The analysis of this question showed that current simulation software can generate simulative parameters and that the SPI evaluation index can evaluate melt quality through experiments. However, both aspects show deficiencies in the ability to predict melt quality on a simulative basis.
2. In the Planning phase, decisions are made about how to achieve the goal [23,25]. Thus, the objective of the planning phase was to analyze the correlation between the input parameters of the SPI and the values generated by a pure simulation.
3. The Data collection phase focuses on collecting, managing, and cleaning the data. It is important to have access to valid and reliable data in order to draw sound and reliable conclusions [23,25]. The data collection includes the SPI data and the problem definition characteristics of the simulation software. The labeled data are used to generate a model for simulating melt quality using machine learning.
4. The Analysis phase involves a series of steps, including organizing data in a structured way, creating tables and graphs to analyze data and calculate predictions, examining data in depth, identifying patterns, conducting planned and unplanned analyses, and formulating hypotheses [23]. In the analysis step, the available data set must be transformed into a processable structure and divided into training and test data. The decision was made to use 10-fold cross-validation, which will be discussed in more detail in the following Section 3.2.
5. The final phase of the PPDAC cycle is Conclusion, which involves careful interpretation of the results and formulation of sound conclusions [23,25,26]. The evaluation and reflection of the model are discussed in Section 4.

### 3.2. Cross-Validation Statistics

One aspect of data preprocessing in machine learning that concerns the structured organization of data is the division of the data set into training and test data. The training data contain the data used to train and optimize the learning model, while the test data are used to evaluate the final model [20]. When dividing a data collection into training and test data, it is important to note that defining test data withhold valuable information from the learning algorithm.

An established method for splitting data sets is cross-validation, which is described in more detail below. In practice, 10-fold cross-validation is often used [27]. In 10-fold cross-validation, the entire data set is randomly divided into 10 equally sized subsets. In each of the 10 estimations, the model parameters are estimated using nine subsets as the estimated data set, while the excluded portion serves as the test data set. The model is validated again by predicting the observations for this part and calculating the prediction error. In this procedure, the estimated total forecast error is the average of the individual 10 errors [27].

### 3.3. Labelling

The input data are the data generated by the simulation software. These are maximum pressure, melt vortex formation, melting length, melt temperature, drive power, minimum residence time, average residence time, and back pressure. The label of the model is the SPI to be determined by the predictive model.

## 4. Model Training and Assessment

**Figure 5:**
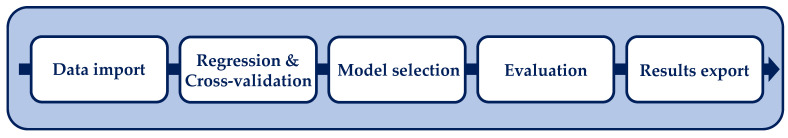
Structure—algorithm.

The task of the Python algorithm is to generate a simulative model from the given data set, which can be used to generate predictions for new process parameters. The source code can be broken down into five steps: importing the data, running the regression with tenfold cross-validation, selecting the best regression model, predicting the data points and evaluating the accuracy, and finally exporting the results to a data format that can be further processed (Excel file). These individual steps, shown in Figure 5, are described below in more detail below.

First, the necessary Python libraries are imported, including pandas for data processing, numpy for mathematical calculations, and the regression and cross-validation modules from the Scikitlearn library. An Excel file containing the data is then read and stored in a Pandas DataFrame. The data include the input variables and an output variable. The input variables are extracted from the DataFrame as a matrix of values and the output variable is extracted as a vector.

**Figure 6:**
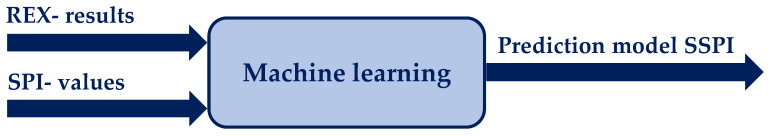
Schematic process of the model.

The REX simulation results are used as input variables in this model (x_1_: maximum pressure, x_2_: melt vortex formation, x_3_: melting length, x_4_: melt temperature, x_5_: drive power, x_6_: minimum residence time, x_7_: average residence time, x_8_: back pressure). The target variable is the experimentally determined SPI associated with these simulations, as shown schematically in Figure 6. No material-specific input variables are required for this model, as these are automatically taken into account by the simulation results.

### 4.1. Regression, Cross-Validation, and Significance Analysis by Lasso Regression

After preprocessing the data, the polynomial regression method is applied. The scikit-learn library is used, in particular the PolynomialFeatures function, to add quadratic terms of the input variables. In preliminary studies, models such as decision trees, the Gaussian process, the gradient boosting model, linear models, and neural networks were trained on the data set. In the validation, the gradient boosting model proved to be the best model with a correlation of 0.86. However, this is a black box model, and the results are difficult to interpret. Since the linear model did not lead to a significantly worse correlation and the analytical equation can be extracted from the linear model, a linear model was chosen. The analytical equation is needed, especially for the implementation of the prediction model for the REX 17.0 simulation software. This equation is used to predict the SSPI based on the REX simulation results.

In order to develop the best model, linear regression models with polynomial terms up to the third order were first created to identify the polynomial with the highest accuracy. Cross-validation can then be performed to evaluate the third-order polynomials. Since the second-order model gives the lowest error, the linear model with second-order terms was selected.

A 10-fold cross-validation was chosen for this model, as this number of folds is seen as a good compromise in the data mining community [28]. This allows predictions to be made based on using 90% of the data. This increases the probability that the resulting model generalizes to the entire data set [28]. In this way, the model is trained and tested on different subsets of the data, providing a more robust evaluation of the model. Another advantage over the hold-out method is that the data set is randomly divided into training and test data. The 10-fold cross-validation leads to a better estimation of the model due to the different subsets of training and test data. In addition, cross-validation averages the results of 10 runs, which reduces the scatter of the evaluation. In this way, overfitting can be counteracted. In general, k-fold cross-validation is a de facto standard in machine learning [29].

The basis for evaluation is the median of the root mean square error (RMSE) of the validation data set [30]. This quantifies the average deviation between the actual and predicted values in a regression model. Unlike the average RMSE, the median RMSE calculates the median of the RMSE values across all convolutions of the cross-validation. The RMSE (Equation (3)) is calculated by taking the squares of the errors between the actual and predicted values, averaging them, and then taking the square root of the result. The median RMSE is the average of the RMSE values when they are sorted in ascending order and the middle (or median) position is reached. The median RMSE is particularly useful when the RMSE values can be strongly influenced by outliers because the median is less susceptible to outliers than the average. Therefore, the median RMSE is often a more robust measure of the performance of a regression model, especially when the error distribution is not normal or when there are outliers in the data. Equation (3) is used to determine this coefficient, where *y*_*real,i*_ is the actual value of the *i*-th data point, *y*_*Prediction,i*_ is the predicted value of the *i*-th data point, and *n* is the number of data points [31].

(3) $$RMSE=\sqrt{\frac{1}{n}\sum_{i=1}^{n}{(y}_{real,i}-y_{Prediction,i}{)}^{2}}$$

The RMSE and median RMSE values are calculated for linear regression models of the first to third order. Since a lower value indicates a better fit than a higher value, the second-order polynomial was chosen. Linear and quadratic terms, as well as interactions among input variables, are included in the model.

To evaluate the model, the correlation between the actual values and the predicted values is also evaluated. Equation (4) can be used to calculate the correlation of a data set. Here, *x*_*i*_ stands for the input parameters, $\bar{x}$ for their mean value, *y*_*i*_ for the target variable, and $\bar{y}$ for its mean value. The covariance of the variables is determined in the numerator, and the product of the standard deviation of the input and target variables is determined in the denominator [32].

(4) $$COR_{xy}=\frac{\sum_{i=1}^{n}\left(x_{i}-\bar{x}\right)*(y_{i}-\bar{y})}{\sqrt{\sum_{i=1}^{n}{\left(x_{i}-\bar{x}\right)}^{2}}*\sqrt{\sum_{i=1}^{n}{\left(y_{i}-\bar{y}\right)}^{2}}}$$

A significance analysis is then performed, which progressively removes less important terms in loops and evaluates the performance of the model using renewed cross-validation. This is done to determine how the performance of the model changes as fewer features are considered. In the end, the linear, quadratic, and interaction terms that provide the best performance are selected.

Significance analysis can be used to reduce the complexity of the data set and thus affect the accuracy of the predictive model by helping to reduce overfitting and improving the generalization ability of the model. Overfitting occurs when a model focuses too much on the details and variations in the training data set. As a result, the model loses its ability to make accurate predictions on new, unknown data. By reducing complexity, potentially misleading or unimportant features are removed. This allows the model to make more robust and reliable predictions on new data by focusing on relevant relationships with the target variable. This has been shown, for example, for image analysis models (e.g., see [33,34].

In this work, a special type of model optimization was chosen, in which a significance analysis is automatically performed and the model is optimized in this way. The linear regression models can be adjusted in several ways to prevent overfitting of the model. Regularized regression is an important technique in machine learning and is used to improve the performance of regression models. There are two commonly used types:

- Ridge regression, which adds the square of the regression coefficients to the regression equation. This stabilizes the coefficients and prevents them from taking on very large values. Equation (5) shows the calculation, where the left term is the squared error between the observed and predicted values, and the right regularization term limits the size of the regression coefficients. The larger α is, the stronger the regularization applied [35]:
  (5) $$Y=\sum_{i=1}^{n}{\left(y_{i}-\hat{y_{i}}\right)}^{2}+\alpha \sum_{j=1}^{p}{\;\beta_{j}}^{2}$$
- Lasso regression, in which the absolute value of the regression coefficients is added to the regression equation. An advantage of lasso regression is that it can reduce coefficients to zero. Automatic feature selection is performed by eliminating the irrelevant variables. The calculation of lasso regression is shown in Equation (6), where the variables are the same as in ridge regression. The difference is in the regularization term, where the absolute values of all regression coefficients are summed [35].
  (6) $$Y=\sum_{i=1}^{n}{\left(y_{i}-\hat{y_{i}}\right)}^{2}+\alpha \sum_{j=1}^{p}\left|\beta_{j}\right|$$

To determine melt quality, the model is constructed using lasso regression.

### 4.2. Model Development Challenges

The main challenge in developing the model was the small number of data points. The generation of these data takes an enormous amount of time, especially due to the experimental investigations to determine the SPI. Therefore, it was not possible to create a large database for this work. In the future, a system will be developed to automatically record and evaluate the experimental investigations of all processes in the KTP laboratory. In this way, additional data points can be generated more quickly, and the model can be further improved.

With regard to the methods of machine learning, the size of the data set plays a decisive role. The less data that can be used to train the model, the less accurate the model will be. The larger the data set, the more data points can be used to validate the model. Before machine learning was considered for this problem, the results were analyzed using traditional evaluation methods such as statistical methods in a tool called “Design Expert”. However, the results were inconclusive. The data did not allow for any meaningful conclusions to be drawn about correlations. To model the data using machine learning, all REX simulation results were used first, so as not to miss any correlations. Significance analysis was then used to identify the number of terms that lead to greater error in the model. Significance analysis also reduces the need to memorize the data, known as overfitting. The more terms that are provided to the model, the more accurately the model can represent the training data. When new data points are used for validation, the model with more terms performs worse.

### 4.3. Results

The model was based on a data set of 74 data points with the simulated results as input variables and the experimentally determined SPI as the output variable. After optimization by lasso regression with α = 0.005, the model yielded the following Equation (7) as the best model for SSPI with 19 coefficients. The value of alpha indicates the strength of regulation of the model. A value of 0.005 was proven to be a good compromise between model complexity and correlation, as well as RMSE and MAE.

(7) $$\begin{array}{cc} SSPI=0.6692 & -0.008\;*\;X_{2}-0.001\;*\;X_{3}-0.012\;*\;X_{4}-0.076\;*\;X_{5}\\ & +0.001\;*\;X_{7}+0.01*\;X_{8}-0.003\;*\;X_{1}\;X_{4}-0.001*X_{1}\;X_{5}\\ & +0.003*{X_{2}}^{2}-0.007\;*\;X_{2}\;X_{3}-0.016\;*\;X_{2}\;X_{4}\;+0.007\\ & *\;X_{3}\;X_{4}\;\;\;\;\;\;\;\;\;\;+0.002\;*\;X_{3}X_{5}\;\;+0.004\;*\;X_{3}X_{7}-0.001\;*\;X_{4}X_{5}\\ & -0.012\;*\;X_{4}X_{8}+0.004\;*\;X_{5}X_{7}-0.005\;*{X_{6}}^{2}\; \end{array}$$

with the input variables *X*_1_: maximum pressure, *X*_2_: melt vortex formation, *X*_3_: melting length, *X*_4_: melt temperature, *X*_5_: drive power, *X*_6_: minimum residence time, *X*_7_: average residence time, *X*_8_: back pressure.

**Figure 7:**
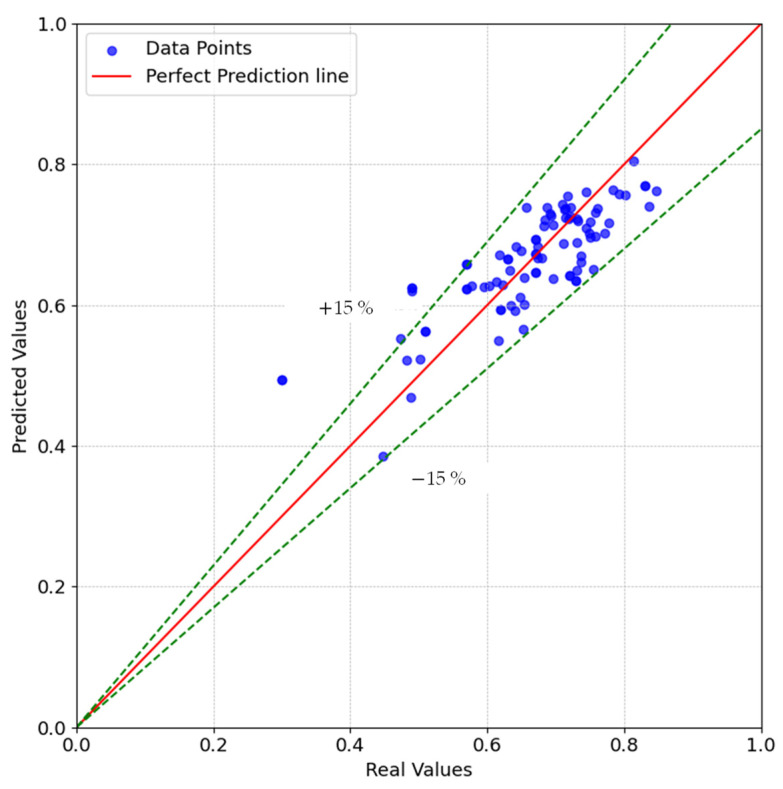
Comparison of predicted SSPI (*y*-axis) and experimental SPI (*x*-axis) for the linear regression model.

Figure 7 compares the predicted SSPI values with the actual SPI values of the data set and shows that the regression equation approximated almost all of the actual SPI values. The deviation of the forecasts was plus/minus fifteen percent. The correlation was 0.824. The RMSE for the optimized model was 0.04 and the MAE was 0.032. The evaluation of the model showed that the prediction error was very low.

The gradient boosting model was also initialized, and the results were compared to classify the prediction quality of the linear model. The gradient boosting model is usually based on the rules of decision trees. For this purpose, a simple base model is first initialized, which introduces errors between the actual and predicted values. New trees are created in multiple iteration loops, continuously reducing the error. The gradient boosting model has been proven to be very effective in modeling complex nonlinear relationships in the data [36]. The RMSE of the initialized gradient boosting model was 0.05, and the MAE was 0.035. Compared to the linear model, the error of the gradient boosting model is larger. For a final evaluation, a validation is required, which is performed below. 

## 5. Validation

The model was also validated with four new experiments that were not used to train the model. Since very few data points are available for validation due to the enormous effort involved in generating the data, only the RMSE and MAE are suitable for model evaluation, and not the correlation. The validation of the model resulted in an RMSE of 0.077 and a MAE of 0.074. The model validation shows that the model predicted the new data with good accuracy.

**Figure 8:**
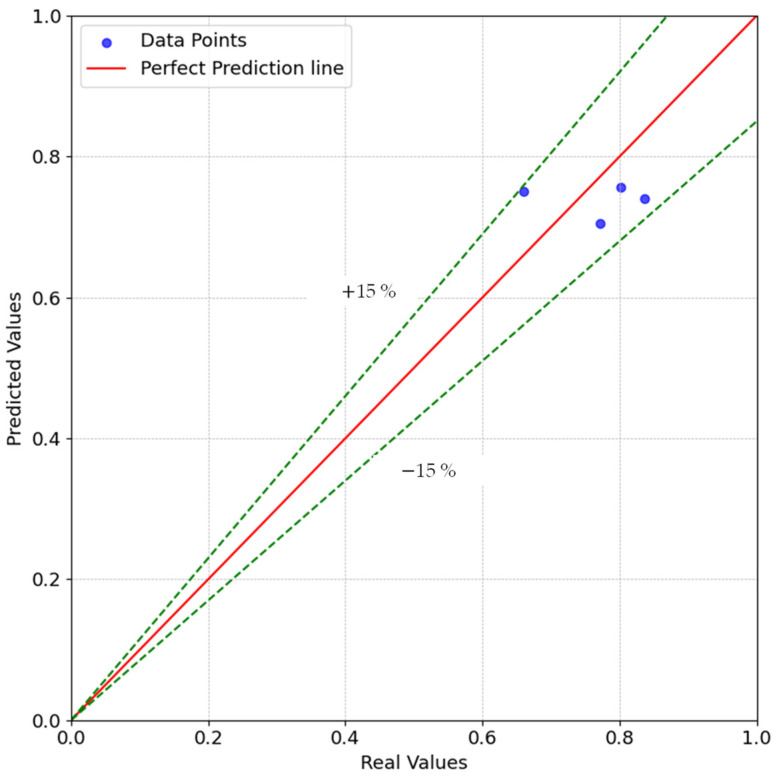
Scatter plot of the validation for the linear regression model.

The scatter plot in Figure 8 shows that the data points did not deviate more than 15% from a perfect prediction. 

The gradient boosting model was also validated using the data set. The calculation of the RMSE showed an error of 0.05, and the MAE, an error of 0.029. Compared to the linear regression model, the gradient boosting model performed better in the validation. This means that this model leads to a better prediction quality. However, since the gradient boosting model is a black box model and no analytical equation can be output, it is not suitable for the application envisaged in this thesis.

Further studies will investigate whether there are more of these outliers and whether the melt quality is also overestimated by the model. This will determine what actions need to be taken to further optimize the model.

However, more data points are needed to conclusively evaluate the model. In addition, different materials and screw diameters should be included in the validation. This will be part of the KTP’s work in the future.

## 6. Conclusions and Outlook

There are numerous definitions of melt quality in the literature, none of which are mutually exclusive, but rather interdependent. Extensive research is required to evaluate melt quality. In order to minimize the amount of research required, this thesis developed a predictive model of melt quality based on the simulation results of the REX extrusion process simulation software. Previous work has already investigated how minimal changes in screw geometry affect melt quality. The evaluation of these investigations shows that the melt quality is influenced, but to such a small extent that this influence is negligible for the prediction model to be developed. In addition, correlations between the thermal and material homogeneity of the melt and Dörner’s SPI were investigated. The results show that the thermal and material homogeneity of the melt correlated with the qualitative index SPI. The more homogeneous the thermal and material distribution of the melt, the higher the resulting SPI. The conditions for the prediction model to be developed in this thesis were derived from the previous investigations: The model should be independent of the screw geometry and the material used. In addition, the experimentally determined SPI can be used as a target value for the melt quality. In order to avoid extensive experimental investigations, the input variables are the simulation results of the REX simulations. Supervised machine learning was used as the modeling method. A linear regression model is suitable for training to obtain an analytical equation. Using 10-fold cross-validation and significance analysis of the coefficients by optimizing the model using lasso regression, a regression equation was developed that predicts the simulated SPI (SSPI). The model achieved a very good correlation between the original data and the predicted melt quality. 

The limitations of this approach lie in the provision of a large data set. Dead stop tests are extremely time consuming and cannot be performed and evaluated during the process. This means that material homogeneity cannot be determined during the manufacturing process. A method would have to be developed to evaluate, for example, the melt during the ongoing film production process, detecting defects as they exit the mold and assessing whether this material homogeneity is sufficient to achieve adequate product quality. Thermal imaging cameras or heat sensors must also be used to evaluate thermal homogeneity. However, this approach only makes sense for the existing process. The predictive model developed in this paper aims to evaluate melt quality during the design process using simulation data.

The model developed for the SSPI needs to be thoroughly validated in the future. In particular, different materials, screw designs, and screw diameters need to be investigated. Limits of the SSPI should then be determined, above which the melt quality is insufficient, and the user can react accordingly during the simulation. The quality of the model should then be evaluated and adjusted if necessary.

By using the predictive model developed in this thesis, manufacturers in the plastics industry do not need to conduct extensive experimental melt quality testing to ensure good product quality for their customers. The user can save a lot of time, energy, and cost by first simulating the extrusion process according to the customer’s requirements and then evaluating the resulting melt quality based on the simulation results. At this stage of screw design, changes in screw geometry, material, and process parameters can be easily made.

The potential of artificial intelligence in mechanical engineering is enormous, especially in the plastics processing industry, where it can bring enormous benefits. Based on this work, further models should be developed that do not refer to the melt quality but directly evaluate the plastic products in the manufacturing process and automatically regulate the process parameters of the process according to the quality.

## Data Availability

Data are contained within the article.
